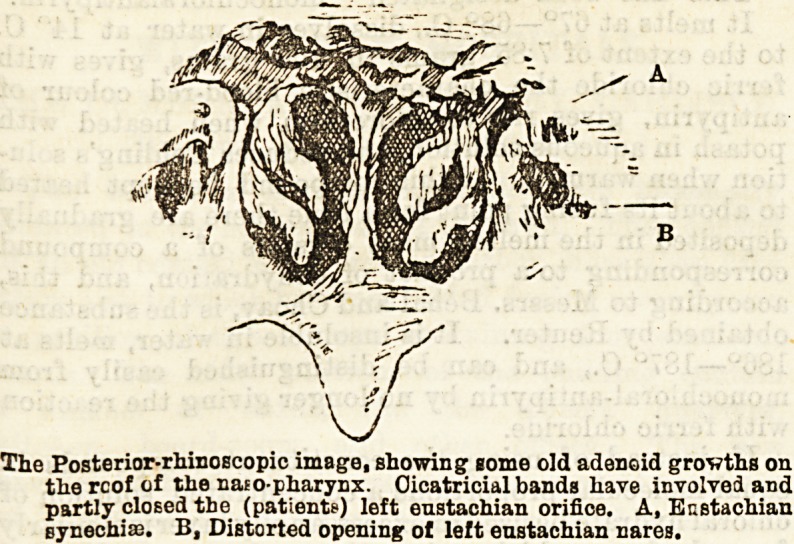# Treatment of Otorrhœa (Discharge from the Ear)

**Published:** 1893-02-25

**Authors:** 


### NEWCASTLE-ON-TYNE THROAT AND EAR HOSPITAL.

### Treatment of Otorrhœa (Discharge from the Ear).

Occurring as this does so frequently in the young and old alike, a general survey of structures contiguous to the ear is first made. The meatus is first cleared of debris and discharge, and its walls examined for ulceration, furunculosis, or diseased bone. The state of the drumhead is now observed, and the position of the perforation and its extent passed in view. If the hole in the drum is high up, the gravity of the condition is noted, as are any granulation tissue or polypi present. Notice is now drawn to the state of the soft parts over the mastoid process, whether these are inflamed or not. The facial expression of the individual is noted in passing, more especially as to whether he is or is not a mouth breather. The tonsils are now passed in review, and indications of granular disease of the pharynx sought for, while by passing the finger behind the soft palate the condition of the postnasal space is determined. In every case of otorrhœa in children it may be said that there will be found present hypertrophy of the tonsil there located, viz., the pharynx tonsil. It may be interesting here to refer to a condition originally described by Dr. Robertson, viz., the fixation of the mouths of the Eustachian tubes by adhesions passing between these and neighbouring parts anchoring these structures and materially interfering with their physiological function. These adhesions Dr.

The Posterior-rhinoscopic image, showing some old adenoid growths on the roof of the naso-pharynx. Cicatricial bands have involved and partly closed the (patients) left eustachian orifice. A, Eustachian synechiæ. B, Distorted opening of left eustachian nares.

Robertson has been able frequently to demonstrate as arising from masses of the pharynx tonsil overlying the tubes becoming organically united to these in course of time. When contraction or atrophy of the tonsil sets in as age advances these adhesions shrink, and correspondingly deform the aspect of the mouths of the Eustachian tubes. Adhesions of a like nature are frequently found at the inferior aspects of the mouths of the tubes, arising from adhesions resulting from masses of lateral granular disease of the pharynx crowding up upon and becoming subsequently adherent to the substance of the mouths. These adhesions, which Dr. Robertson has called "Eustachian synechiae," are met with not earlier than the age of twenty years, and are, without doubt, a frequent cause of tubal catarrh. In no text-book have these conditions been noted, although in the schools they are referred to, demonstrated, and due weight accorded them as etiological factors in various forms of ear disease.

In every case of chronic otorrhœa, after careful inquiry into the history of its occurrence, duration, etiology, &c., attention is directed towards determining as to whether or not there are any brain symptoms. More especially are the slighter forms of such carefully looked for, viz., giddiness, vomiting, deep-seated pain in the ear, &c.; in fact, such symptoms as would point to more or less severe irritation of the dura mater. A deep red, angry-looking state of the soft parts situated in the neighbourhood of the drumhead is taken as an indication in chronic otorrhœa, that grave necrotic mischief is existent in the petrous portion of the temporal bone. Together with the above symptoms it becomes a near certainty.

Amongst the rarer factors producing or perpetuating otorrhœa, there may be mentioned polypus of the nose, ozena, and suppuration of Highmore's autrum. Of causes in the external meatus, reference may be made to bony growths there, and at rare intervals foreign bodies in the canal.

In the case of children troubled with otorrhœa, so frequently is enlarged pharynx tonsil present that the post-nasum is at once explored, and the size and extent of the growth made out. The treatment therefore begins with the removal of this growth. As preliminaries to its removal, the nasal douche is used, generally composed of a solution of boracic acid, care being taken that no reflux of fluid takes place into the Eustachian tubes. Then a syringe (with a long canula) filled with a solution of 2 per cent. each of resorcin and cocain is introduced through each naris into the post-nasum, and a short interval allowed for the cocain to act. The most rapid and least dangerous instrument with which to take off this growth is Gottstein's heart-shaped curette, and that variety of it which has the broad part of the curette slightly curved towards its handle, so as to fit the roof of the post-nasum. Chloroform is dispensed with, and, choosing the size of curette suited to the case, it is introduced behind the soft palate. It is now pressed well on to the growth, and made to scrape it off from the mucous membrane. On bringing out the instrument the growth often follows, clinging to the curette. The child's head is now held over a basin, so as to allow any blood to escape. The next and most important step of the operation is effected by the finger, and that is to introduce it and remove remains of the growth, which are often found occluding the posterior nares and preventing nasal respiration, and fragments found lodged in parts surrounding the mouths of the Eustachian tubes, and thus liable to give rise to Eustachian synechiæ if left behind. As to the question of giving chloroform in this operation, in the practice of the hospital it is as a rule dispensed with, provided that the child is of ordinary physique and fairly nourished. Nor is it considered that thereby the growth is imperfectly removed.

In older patients a careful post-nasal examination, either with the post-nasal mirror or the finger, is made, not forgetting the fact that, contrary to the teaching of the text-books, an enlarged pharynx tonsil is often found, even up to sixty years of age. In adults the frequency of Eustachian synechiæ is recognised, and these, if found, are ruptured and scraped off by introducing the finger to their site, and sweeping it round the tubes and thus freeing them.

Systematic treatment of the post-nasum is maintained for some weeks after by nasal douches and local swabbing of the space with Lugol's solution,* effected by means of cotton wool on a wool-holder, to the end that the general catarrh of the mucous membrane of the post-nasum and of the mouths of the Eustachian tubes in every case present may become resolved.

To overlook the treatment of these parts in a case of otorrhœa is merely to court failure. In a large number of cases the means above recorded often suffice to check the ear disease and restore hearing. Attention, of course, is given to the purely local condition in the ear. As an ear wash, a 25 per cent. solution of spirits of wine, used to syringe out the ear, to be followed afterwards by lightly blowing in some powdered boracic acid is ordered. But to this we shall return.

## Figures and Tables

**Figure f1:**